# Physics of sliding on water explains morphological and behavioural allometry across a wide range of body sizes in water striders (Gerridae)

**DOI:** 10.1098/rspb.2024.1357

**Published:** 2024-12-18

**Authors:** Woojoo Kim, Jae Hong Lee, Thai Hong Pham, Anh Duc Tran, Jungmoon Ha, Sang Yun Bang, Jeongseop Lee, Piotr G. Jablonski, Ho-Young Kim, Sang-im Lee

**Affiliations:** ^1^ School of Biological Sciences, Seoul National University, Seoul 08826, Republic of Korea; ^2^ Institute of Biodiversity, Seoul National University, Seoul 08826, Republic of Korea; ^3^ Research Institute of Basic Sciences, Seoul National University, Seoul 08826, Republic of Korea; ^4^ Department of Mechanical Engineering, Seoul National University, Seoul 08826, Republic of Korea; ^5^ Mientrung Institute for Scientific Research (MISR), Vietnam National Museum of Nature, Vietnam Academy of Science and Technology (VAST), 321 Huynh Thuc Khang Street, Hue, Vietnam; ^6^ Graduate University of Science and Technology, VAST, 18 Hoang Quoc Viet, Cau Giay, Hanoi, Vietnam; ^7^ Department of Applied Zoology, Faculty of Biology, University of Science, Vietnam National University, 334 Nguyen Trai, Thanh Xuan, Hanoi, Vietnam; ^8^ Museum and Institute of Zoology, Polish Academy of Sciences, Warsaw, Poland; ^9^ Institute of Advanced Machines and Design, Seoul National University, Seoul 08826, Republic of Korea; ^10^ Laboratory of Integrative Animal Ecology, Department of New Biology, DGIST, Daegu, Republic of Korea

**Keywords:** sliding, allometry, water strider, water surface, surface tension, locomotion

## Abstract

Laws of physics shape adaptations to locomotion, and semiaquatic habitats of water striders provide opportunities to explore adaptations to locomotion on water surface. The hydrodynamics of typical propelling with symmetrical strokes of midlegs is well understood, but the subsequent passive sliding on surface has not been explored. We hypothesized that morphological and behavioural adaptations to sliding vary by body size. Based on empirical observations of water striders across a wide range of body size, we constructed a theoretical model of floating and resistance during sliding. Our model predicts that large water striders are too heavy to support anterior body on forelegs during sliding when their two midlegs are off the surface symmetrically during a recovery phase after stroke in symmetric gait. Heavy species should either (i) develop sufficiently long forelegs to support their anterior body on surface during symmetric gait or (ii) use asymmetric gait when one forward-extended midleg supports anterior body. Observations were consistent with these predictions. Additionally, medium-sized insects were observed to switch between symmetrical and asymmetrical gait in the manner that reduces sliding resistance. Our results illustrate how habitat-specific physical processes cause morphological and behavioural diversity associated with body size among biological organisms.

## 1. Introduction

Allometry, the study of how physics and biology affect the relationships between body size and other characteristics of an organism, has a long history [1–10] and is of great importance not only to biology [11–13] but also to the modern bioinspired engineering [14,15]. Distinguishing between specific biological and physical mechanisms/constraints responsible for allometry may often be challenging [4,12]. However, some organisms offer a good study system where allometry can be attributed to physical constraints. Animals that live on the water surface are exposed to specific physical constraints from the water surface, and it has been suggested that body size affects morphological and behavioural adaptations to semiaquatic locomotion in animals [16–20]. Water striders of the family Gerridae are ideal subjects for testing this idea. Although many studies have taken theoretical approach to understand the physics of water striders’ locomotion [18,19,21–36], the research effort is confined to several small- and medium-sized species while water striders’ body mass spans over two orders of magnitude from less than 5 [37] to about 500 mg [38]. While precedent studies focused on propelling mechanism, our research focuses on how body size and leg morphology affect the sliding locomotion of water striders.

The typical locomotion of Gerridae comprises the ancestral symmetric gait of striding/skating (electronic supplementary material, video S1) [30,39,40], in which midlegs symmetrically push backwards (propulsive phase) to create forward movement of an insect while its body is supported on the water surface by two forelegs and two hindlegs for the duration of the push and the subsequent sliding (on the surface) or leaping (above the surface) until the midlegs return to their original positions (recovery phase) on the water surface and braking occurs (short-lasting braking phase). The anterior body section remains supported only on short forelegs when the insect passively slides after propelling. The heavier the body the stronger the capillary force from forelegs is required for support. If this force is too large, the surface under the short forelegs breaks and the insect cannot stay afloat. Hence, floating on the surface during passive sliding (floatability) is the first theoretical consideration in predicting locomotive adaptations in large-bodied water striders. Additional considerations are the effects of body mass, wetted leg length and sliding velocity on resistance that legs experience on the water surface [41–43]. Following the convention in the literature, we use the term ‘wetted length’ as water-contact length even though the leg is not technically ‘wet’ due to its hydrophobicity. The resistance causes deceleration during sliding and affects the sliding performance such as the distance reached during a single slide. Furthermore, the resistance experienced by an insect can affect the aforementioned floatability. This is due to the tail-up pitching torque generated by the resistance, which results in an additional downward force to the insect’s forelegs.

Several observations from the literature suggest that heavy water striders evolved unique foreleg morphology and/or gait to support the anterior part of the body on water. First, disproportionately elongated wetted forelegs in large-bodied water striders of the genus *Ptilomera* [39,44] may help to support the anterior body section during propelling and passive sliding. However, as this type of morphology is also observed in small species of Gerridae (e.g. in Halobatinae [22,37]), it may not necessarily be a specific adaptation to heavy body, but rather to their midlegs not being used for support on the water surface (ascertained for Ptilomerinae [44]). Second, asymmetric gait that involves one midleg extended forward to support the heavy anterior body part while the other midleg provides propulsion may be a specific adaptation to heavy body. This locomotive behaviour was only reported in the world’s largest water strider species, *Gigantometra gigas*, also known as the giant water strider (electronic supplementary material, video S1) [38]. In this asymmetric gait, forelegs may not be crucial for the anterior body support, and *G. gigas* has relatively short forelegs [38,45].

**Figure 1.**
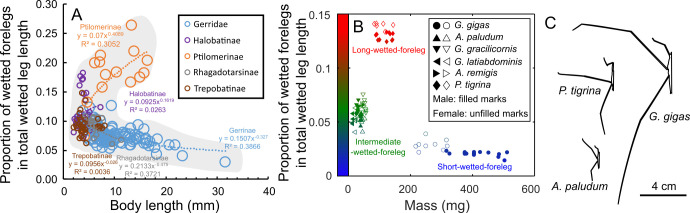
Leg morphology (‘leg geometry’) across Gerridae, and body length and mass of the study species. (A) Body length and leg proportions across Gerridae [45]. (B) Body mass and leg proportions of studied species. (C) Silhouettes of three species at the same scale. Leg morphology is expressed as proportions of wetted forelegs in the total wetted length of six legs of an individual water strider (‘wetted leg geometry’). Species with ‘intermediate-wetted-foreleg geometry’: *Aquarius paludum*, *A. remigis*, *Gerris gracilicornis* and *G. latiabdominis*. Leg geometry of *A. paludum* (green triangles) was used as the representative of ‘intermediate-wetted-foreleg geometry’ (approx. 4–8%), *Ptilomera tigrina* served as the representative of ‘long-wetted-foreleg geometry’ (approx. 12–14%) and *Gigantometra gigas* served as representative of the ‘short-wetted-foreleg geometry’ (approx. 1–3%) in the model. For more explanations and data, see captions to electronic supplementary material, figures S1–S5.

Based on these observations, and on a brief review of morphological measurements of Gerridae from the literature (figure 1A; electronic supplementary material, figures S1 and S2), we introduce a concept of ‘wetted leg geometry’. Considering the proportion of wetted forelegs in the total wetted length of all six legs, a species can be classified into three types of ‘wetted leg geometry’: a ‘short-wetted-foreleg geometry’ (extremely developed in *G. gigas*), an ‘intermediate-wetted-foreleg (or “standard”) geometry’ observed in the frequently studied small- and medium-sized genera of *Gerris* and *Aquarius*, and a ‘long-wetted-foreleg geometry’ (e.g. in Halobatinae, Ptilomerinae; electronic supplementary material, figure S1 shows ranges of values for different taxa). Data extracted from the literature suggest two trends associated with an increase in species body size: elongation or shortening of forelegs in Gerridae (figure 1A; electronic supplementary material, S2A).

Support for the anterior body part and the resistance on the legs during sliding should be both considered to predict feasible combinations of the ‘wetted leg geometry’ and gait (symmetric or asymmetric) that prevent breaking of the water surface by a water strider of a given body mass. Here, we empirically study morphology and sliding behaviour in several species that represent the three ‘wetted leg geometries’, and we develop a theoretical model of the hydrodynamics of passive sliding (recovery phase) in symmetric and asymmetric gaits for the three types of wetted leg geometry across a wide range of the water strider body size.

## 2. Results

### (a) The ‘wetted leg geometry’ of the studied species

Although individuals from the six study species followed a general relationship between body mass and the total wetted leg length (electronic supplementary material, figure S4), they differed in the relative proportions of wetted forelegs (figure 1A,B), midlegs and hindlegs (figure 1C; electronic supplementary material, figure S5). It is important to consider this proportional difference because forelegs support the insect’s frontal body during symmetric sliding, and wetted foreleg length can influence floatability as insects slide after a symmetric stroke. Here, the ‘wetted length’ was assumed to be tarsus/tarsus+tibia for foreleg/hindleg based on empirical observations of maximal wetted leg length (electronic supplementary material, figure S14, videos S2–S4, S6 and S7; except for unusual situations in electronic supplementary material, videos S5 and S9). The wetted length of anterior supporting midleg in asymmetric gait is more variable than other legs: half of midleg (tarsus+tibia) in fast sliding of large-sized species (electronic supplementary material, figure S14C–E) and half of tibiotarsal segment (one-half (tarsus+tibia); about 1/4 of midleg) at the minimum in medium-sized species (electronic supplementary material, figure S14A,B).

For the morphological analysis of the ‘leg geometries’ across Gerridae, for which behavioural information is not collected, we used the following definition of the wetted midlegs: the maximum wetted length (tarsus+tibia) that may interact with water during locomotion. We also present results that assume the minimum length of wetted midleg (half of tibiotarsal segment) in electronic supplementary material 1 (for both ‘leg geometry’ and model simulation outcomes, electronic supplementary material, figures S3, S29 and S32). Regardless of the assumption, the 222 species from the literature (electronic supplementary material, figures S2 and S3) and the water striders in this study (figure 1B; electronic supplementary material, figure S5) clearly form three groups: ‘short-wetted-foreleg’, ‘intermediate-wetted-foreleg’ and ‘long-wetted-foreleg’ group.

The four small/medium-sized study species (*Gerris latiabdominis*, *G. gracilicornis*, *Aquarius remigis*, *A. paludum*) formed one cluster of ‘intermediate-wetted-foreleg geometry’ with wetted forelegs comprising from approx. 4% to 8% of total wetted leg length (figure 1B). For theoretical modelling, we decided to use the specific values of the ‘wetted leg geometry’ of *A. paludum* (figure 1B) as the representative ‘intermediate-wetted-foreleg geometry’. The ‘long-wetted-foreleg geometry’ with wetted forelegs comprising 12−14% of the total wetted leg length was represented by *P. tigrina* (figure 1B), and the ‘short-wetted-foreleg geometry’ with wetted forelegs comprising 1−3% was represented by *G. gigas* (figure 1B; electronic supplementary material, figures S4 and S5).

### (b) Empirical observations of locomotion

**Figure 2.**
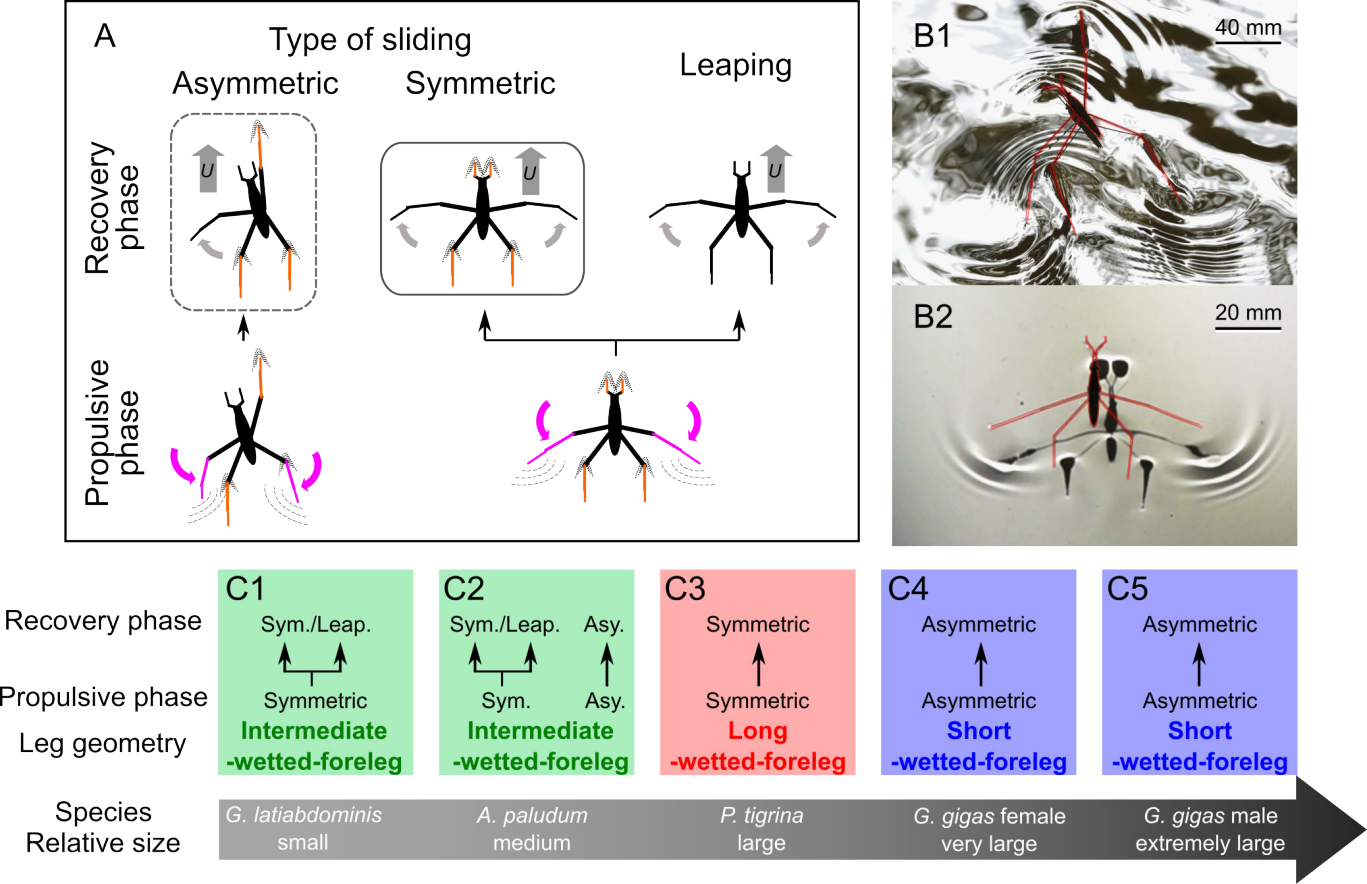
Summary of observations of locomotion of the study species (see electronic supplementary material, videos S1–4,6). (A) Schematics of locomotion modes in water striders. Gait has two phases: propulsive phase (when legs push backwards and create a thrust forward), and recovery phase (when water strider is sliding on water or leaping above water). During the power stroke, the rowing gait could be either symmetric (typical for most Gerridae) or asymmetric. The leaping is preceded by symmetric propulsion. Coloured legs indicate propelling legs (purple) and sliding legs (orange). (B) Two types of propulsion: asymmetric (B1, *G. gigas*) and symmetric (B2, *A. paludum*). (C) Observed locomotion modes. The large grey arrow indicates relative size of species. Leg geometries: ‘short-wetted-foreleg’ (blue), ‘intermediate-wetted-foreleg’ (green) and ‘long-wetted-foreleg’ (red). Water strider size classes: *G. latiabdominis* (C1; body length: 9.6−15.0 mm); *A. paludum* (C2; 14.1−16.0 mm); *P. tigrina* (C3; 15.4−18.7 mm); *G. gigas* females (C4; 31.4−33.9 mm) and *G. gigas* males (C5; 31.8−40.2 mm). Electronic supplementary material, videos illustrate natural gaits of *G. latiabdominis* (electronic supplementary material, video S2), *A. paludum* (electronic supplementary material, video S3), *P. tigrina* (electronic supplementary material, video S4) and *G. gigas* (electronic supplementary material, video S6).

We observed three combinations of propulsive phase and recovery phase: symmetric propelling—symmetric sliding, symmetric propelling—leaping, and asymmetric propelling—asymmetric sliding (figure 2A,B). The smallest species with ‘intermediate-wetted-foreleg geometry’, *G. latiabdominis*, used symmetric propelling—symmetric sliding and symmetric propelling—leaping (figure 2C1; electronic supplementary material, figures S6–S11, video S2). The medium-sized species with ‘intermediate-wetted-foreleg geometry’, *A. paludum*, used all three combinations (figure 2C2; electronic supplementary material, video S3). The large species with ‘long-wetted-foreleg geometry’, *P. tigrina*, used symmetric propelling—symmetric sliding combination (figure 2C3; electronic supplementary material, video S4). Only when forelegs were handling the food [44] or grooming (electronic supplementary material, video S5), *P. tigrina* used asymmetric gait, indicating individual’s ability to switch between gait types. Both sexes of the largest species with ‘short-wetted-foreleg geometry’, *G. gigas*, used only the asymmetric gait (figure 2C4,5; electronic supplementary material, video S6): one midleg always supported anterior part of the body (electronic supplementary material, video S7). In asymmetric sliding of both *A. paludum* and *G. gigas*, the forelegs were observed to either touch the water surface or remain elevated (electronic supplementary material, video S3), and the insects generally did not contact the water with their forelegs during fast sliding (electronic supplementary material, video S6). Two large species, *G. gigas* and *P. tigrina*, did not use leaping.

*P. tigrina* consistently maintained its forelegs parallel to the movement direction (electronic supplementary material, video S4), *G. gigas* also kept the anterior supporting midleg parallel to the direction of sliding (electronic supplementary material, video S6). *A. paludum* exhibited various foreleg tarsus orientations, with parallel, near-parallel and slightly diagonal to the movement direction being the most common situations, while perpendicular orientation was observed occasionally (see electronic supplementary material, figure S17, video S9 and table S2). Additional observations of *G. latiabdominis* suggested that this species may show similar variation in the orientation of forelegs to *A. paludum*.

### (c) Basic concepts in the theoretical model of sliding based on empirical observation

**Figure 3.**
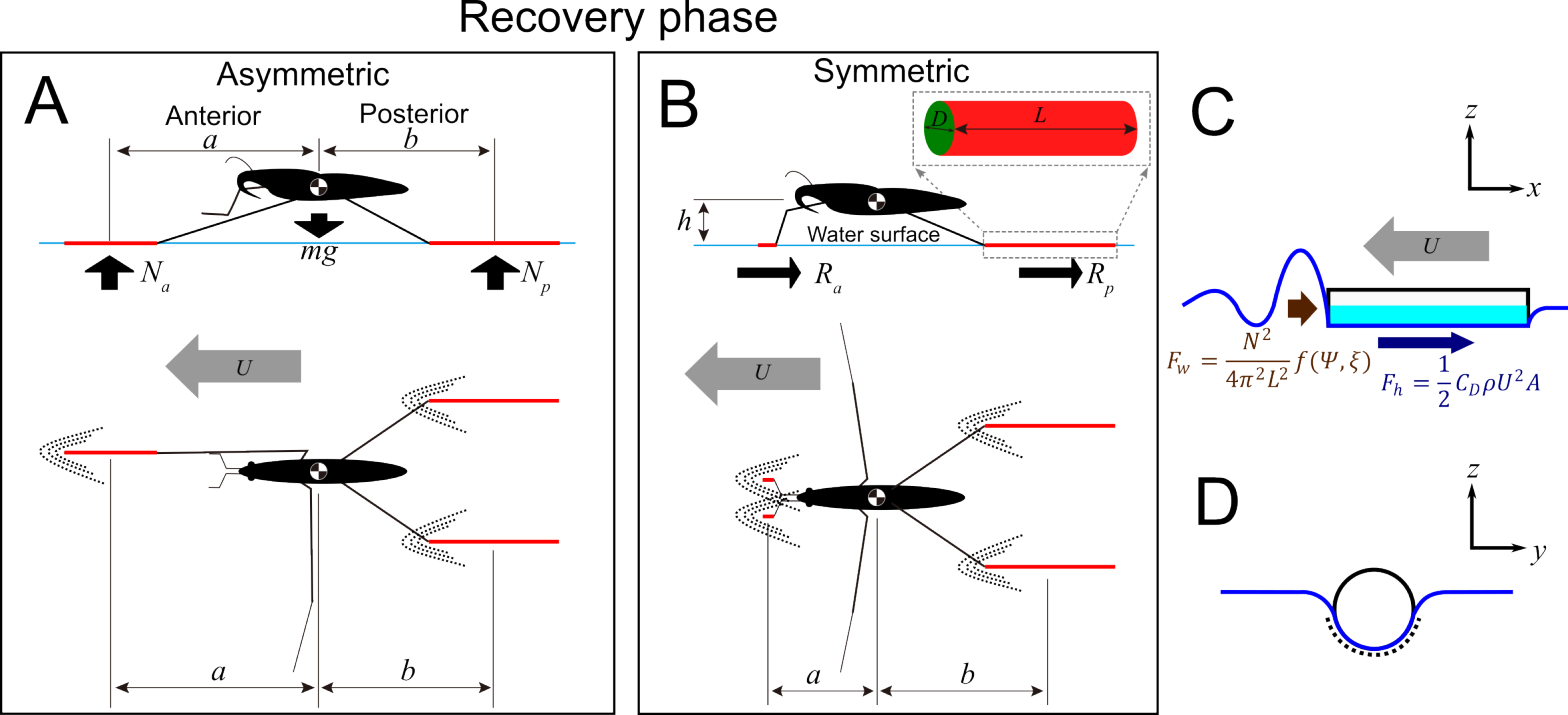
Graphical explanation of the basic concepts in the model of sliding water strider. (A,B) schematics of asymmetric (A) and symmetric (B) sliding, and variables used in the model: anterior and posterior normal forces ($N_{a}$, $N_{p}$), the anterior and posterior resistance forces ($R_{a}$*,* $R_{p}$), wetted leg lengths (L) and diameters (D), horizontal distance along line parallel to the moving direction from the centre of the mass to the centre of the anterior and posterior supporting wetted legs (a*,*b), body height above water surface (h), body velocity (U). (C,D) the two main forces contributing to the total resistance: hydrodynamic drag ($F_{h}$; brown) and wave drag ($F_{w}$; blue). The light blue area (C) and dotted line (D) represent the wetted area of a sliding leg. $C_{D}$ is the surface tension coefficient, ρ is the density of water, U is the relative velocity of the water strider to the water and A is the wetted area of the leg. The wave drag, $F_{w}$, is induced by the wave generated by the cylindrical leg as shown in C. N is the normal force on the leg from the water (A), L is the leg length, Ψ is the shape of wetted area depending on the shape of the leg and its moving direction. ξ is designed to simplify the formula. See details in electronic supplementary material 1, parts 3 and 4 (electronic supplementary material, figures S13–S27).

To understand the observed variability in leg geometry and gait, we built a theoretical model of the sliding (recovery phase). The full details of the model and its empirical validation from observations of the study species are presented in electronic supplementary material 1 parts 3 and 4 (electronic supplementary material, figures S13–S27). Although the model is based on a series of simplifying assumptions, we believe it captures the essence of physical processes during sliding. We model a leg as a smooth-surface cylinder (by ignoring claws of legs; electronic supplementary material, figure S15) with certain length and diameter imitating water strider legs surrounded with hairs [20]. Water striders slide with body velocity, U (relative to the water surface), either symmetrically on two forelegs and two hindlegs or asymmetrically on a midleg and two hindlegs. Therefore, anterior supporting leg(s) can be either two forelegs or one midleg. During sliding (figure 3A,B), normal upward forces (the anterior, $N_{a}$, and the posterior, $N_{p}$, normal force) keep the insect afloat, while resistance on the legs interacting with water (the anterior, $R_{a}$, and the posterior, $R_{p}$, resistance) gradually slows down the passively sliding insect. This sliding resistance generates a torque that presses down the anterior supporting legs, causing an increase of anterior supporting force ($N_{a}$) needed to prevent meniscus breaking. In summary, the model calculates the total resistance of sliding legs, then tests the floatability of a sliding insect by considering its body mass and the torque generated by the resistance.

We consider two types of resistance force [31,46] applied to the water strider legs during passive sliding (see electronic supplementary material 1, part 3): hydrodynamic drag (figure 3C,D) and wave drag (figure 3C,D; explanations of symbols are in caption to figure 3 and in electronic supplementary material 1, part 3, table S1).

We first consider the resistance force on one leg. We assume that a leg is a sliding cylinder oriented parallel to the direction of movement (figure 3A,B), and that half of the surface of the wetted leg interacts with the water surface (electronic supplementary material, figure S15) regardless of the water strider mass. In this typical parallel leg case, the hydrodynamic drag, $F_{h}=\frac{1}{2}C_{D}\rho U^{2}A$, is dominantly caused by the shear stress acting on the wetted area of the leg since the leg is sliding parallel to its longitudinal axis (figure 3C,D) [47]. It is a function of water properties (density, ρ, kinematic viscosity, ν), leg morphology (wetted area, A; the effect of the surface structure was not considered in this study) and water strider behaviour (water strider velocity, U, relative to the water surface).

To reflect the empirically observed variation in anterior supporting leg’s wetted length and orientation during sliding (see above ‘Empirical observations of the locomotion’), we run additional model simulations for two extreme cases: one for minimal length of parallelly oriented anterior leg; one for anterior leg’s orthogonal orientation (see electronic supplementary material, part 3). When a leg is orthogonal to the movement direction, the hydrodynamic drag ($F_{h}$) is dominantly caused by the pressure drag on a sliding leg.

The capillary-gravity wave drag, $F_{w}=\frac{N^{2}}{4\pi^{2}L^{2}}f(\Psi ,\xi )$, is induced by the wave on the waterfront of the cylindrical leg/water interface as shown in figure 3. It occurs at body velocities larger than the critical value *c* = 0.2313 m s^−1^, when a moving water strider creates a visible wave on the water surface (also empirically proven in electronic supplementary material, figure S18). We assumed that this drag is a function of water properties (density, ρ, kinematic viscosity of water, ν, and surface tension coefficient, σ), morphology (normal force, N, leg length, L*,* and shape of wetted area, Ψ) and water strider behaviour (normal force, N, body velocity relative to water surface, U).

We derived a simple gravity-normal force balance formula and torque-balance formula for the posterior, $N_{p}$, and anterior, $N_{a}$, normal forces taking into account the water strider body mass, leg morphology (distances a, b, and wetted leg lengths on forelegs and/or hindlegs; figure 3A,B; electronic supplementary material 1, part 3) and the resistance force on the anterior and posterior supporting legs ($R_{a}$ and $R_{p}$; electronic supplementary material 1, part 3). The larger resistance leads to the larger rate of deceleration, and the heavier the body leads to the lower rate of deceleration.

Finally, from the calculations of the system of equations from the two balance formulae, the model predicts the normal forces, $N_{a}$ and $N_{p}$, the total resistance as a sum of anterior, $R_{a}$, and posterior, $R_{p}$, resistance on water strider legs, and deceleration caused by the resistance during sliding. Hence, we computationally determined $N_{a}$, $N_{p}$, $R_{a}$ and $R_{p}$ (figure 3A,B) for a water strider sliding on the surface in a situation comprising the following set of empirically derived values: body mass, m, wetted leg lengths, L, wetted leg diameters, D, distances a and b (as defined in figure 3A,B), vertical distance between surface and water strider body, h (figure 3B) and body velocity, U, during sliding. Further details of the mathematical model are provided in electronic supplementary material 1, part 3.

### (d) Model predictions for five different size classes and three leg geometries

**Figure 4.**
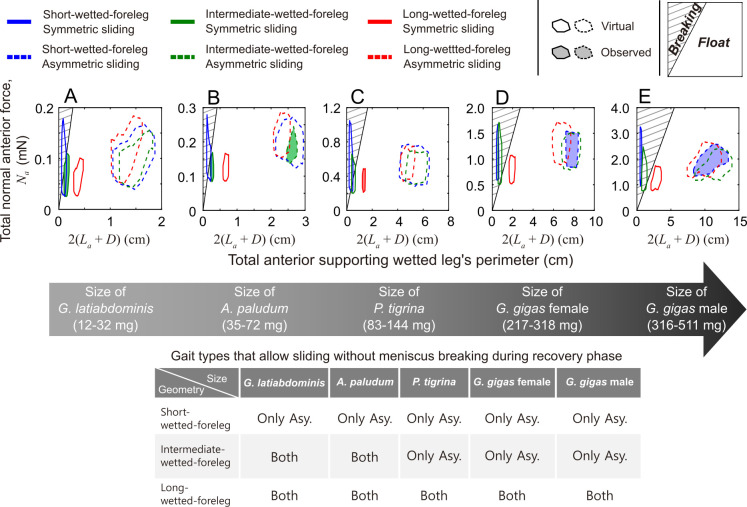
Model predictions of floating conditions. Predictions of ability to float without meniscus breaking during sliding for different combinations of ‘leg geometry’, body size class and sliding mode. The large grey arrow at the middle represents size classes based on our study organisms: *G. latiabdominis*, *A. paludum*, *P. tigrina* and *G. gigas* females and males. Wetted leg geometries: ‘short-wetted-foreleg’ (blue), ‘intermediate-wetted-foreleg’ (green) and ‘long-wetted-foreleg’ (red). Sliding types: symmetric sliding (solid line) and asymmetric sliding (dashed line). Filled polygons represent observed combinations of behaviour and leg geometry; unfilled polygons represent virtual combinations of behaviour and leg geometry for different body sizes. The phase diagrams represent normal force applied on anterior supporting leg ($N_{a}$; vertical axis) and total length of the anterior supporting legs’ wetted perimeter ($2(L_{a}+D)$; horizontal axis). The diagonal black solid line in each figure corresponds to $N_{a}=2\sigma (L_{a}+D)$, and the hatched area above the line indicates conditions leading to meniscus breaking under the anterior supporting leg(s). The table summarizes the floating conditions predicted by the model.

After validating model assumptions (electronic supplementary material 1, part 4) and confirming that the theoretical model reasonably simulates empirically observed trajectories (electronic supplementary material, figures S19–S24)*,* we used it to predict how the three ‘leg geometries’ would perform in terms of floating on the water during sliding, and in terms of resistance and deceleration during symmetric and asymmetric sliding for five body size classes corresponding to the recorded body mass (electronic supplementary material, figures S4 and S5) and velocity ranges (electronic supplementary material, figure S10): *G. latiabdominis* (12−32 mg, 0−1 m s^−1^), *A. paludum* (35−72 mg, 0−1.5 m s^−1^), *P. tigrina* (83−144 mg, 0−2.5 m s^−1^), *G. gigas* females (217−318 mg, 0−2.5 m s^−1^) and *G. gigas* males (316−511 mg, 0−2.5 m s^−1^). This resulted in predictions for 30 situations (5 body mass classes × 2 gait types [symmetric or asymmetric] * 3 ‘leg geometries’) including six situations observed in our study subjects (figure 2C) and 24 ‘virtual’ ones that have not been recorded in our study species (figure 4; electronic supplementary material, figures S29 and S30).

The maximum (critical) force resulting from surface tension acting on anterior supporting leg(s) is the product of surface tension coefficient, σ, and the wetted length consisting of the length, $L_{a}$, and diameter, D (figure 3B); ($2\sigma (L_{a}+D))$ [32,33]. Therefore, the anterior supporting leg(s) would pierce through the meniscus when the total force needed to support the anterior part of body, $N_{a}$, is larger than $2\sigma (L_{a}+D)$. Using the theoretical model, we produced two-dimensional phase diagrams in figure 4, with the anterior normal force, $N_{a}$, on the vertical axis and the wetted leg perimeter ($2(L_{a}+D)$) on the horizontal axis. The conditions when the sliding water strider’s anterior supporting leg(s) do not pierce the water surface correspond to the unhatched area below the line of the critical $\;N_{a}=2\sigma \left(L_{a}+D\right).$ The hatched area above this line comprise situations in which the anterior supporting leg(s) pierce the water surface (i.e. the total normal anterior force is larger than maximum force resulting from surface tension). The model predicted that the water striders do not break the meniscus if they perform asymmetric sliding in any of the 45 conditions defined by three leg geometries and five body size classes (all polygons with dashed edges in figure 4) and the three simulated scenarios (defined by anterior supporting leg length and orientation: electronic supplementary material, figures S29 and S30).

Regardless of the body size, water striders with ‘long-wetted-foreleg geometry’ (red solid line polygons) performing symmetric striding were not predicted to break meniscus provided that they keep their long forelegs parallel to the sliding direction. In this case, maintaining the near-parallel orientation of forelegs is crucial for larger species because the model predicted meniscus breaking in the extreme situation of orthogonal foreleg orientation by large species at high sliding speeds corresponding to larger values of the total anterior normal force (electronic supplementary material, figure S30C–E).

The three larger size classes of water striders with ‘intermediate-wetted-foreleg’ (green solid line polygons in figure 4C–E, electronic supplementary material, figures S29C–E and 30C–E) and any size class of water striders with ‘short-wetted-foreleg’ (blue solid line polygons in figure 4; electronic supplementary material, figures S29 and S30) were predicted to break the meniscus if they perform symmetric sliding except for a very narrow range of conditions that locate them under the critical lines of $N_{a}$ (figure 4A–C, and electronic supplementary material, figures S29A–C and 30A–C for ‘short-wetted-foreleg’; figure 4C–E, and electronic supplementary material, figures S29C–E and 30C–E for ‘intermediate-wetted-foreleg’), which is associated with slower sliding or being at rest (not moving). *G. gigas*, the largest water strider, was predicted to be unable to stay afloat using their short forelegs even in the static condition (e.g. all blue solid polygons are in hatched area in figure 4D,E, electronic supplementary material, figures S29D,E and 30D,E, video S7). These predictions were consistent with the empirical observations (figure 2C): large-sized water striders used asymmetric gait (electronic supplementary material, video S6) or had long forelegs oriented near parallel during symmetric sliding (electronic supplementary material, video S4).

The two smaller species with ‘intermediate-wetted-foreleg’ geometry (*G. latiabdominis* and *A. paludum*) are predicted not to break the meniscus during symmetric gait unless they slide fast and their forelegs are in the atypical extreme orthogonal orientation to the sliding direction when high normal force is needed (green solid polygons in electronic supplementary material, figure S30A,B). The cases of foreleg breaking were indeed occasionally observed in *A. paludum* (electronic supplementary material, table S2, figure S17, video S9) under the laboratory conditions.

In summary, our model predicts that large species are too heavy to support their bodies during sliding when they have leg geometry and gait typical for smaller species. They have to either use asymmetric gait or have elongated forelegs to slide without meniscus breaking (summary table in figure 4; electronic supplementary material, figure S28). Empirical observations are consistent with the model outcome.

### (e) Gait type depends on body speed—behavioural plasticity in small/medium-sized species

Floatability predictions for large-sized species explain the reasons behind their specific sliding gaits and different leg geometries. On the contrary, the medium-sized species, *A. paludum*, was predicted to float during sliding regardless of whether they use asymmetric or symmetric sliding (except for case of fast symmetric sliding with orthogonal foreleg tarsi; figure 4; electronic supplementary material, figures S29 and S30). The presence of both symmetric and asymmetric gait in *A. paludum* was consistent with this prediction (figure 2C; electronic supplementary material, figure S17, video S9). Therefore, we focused on the behavioural plasticity of *A. paludum*. Since the presence of multiple gait types within a single species (or individual) cannot be solely explained by floatability prediction, we hypothesized that insects may choose their gait types depending on the resistance/deceleration. The model predicted that differences between asymmetric and symmetric sliding in deceleration can be considerable in *A. paludum*, especially for high sliding velocities (electronic supplementary material, figures S31G, S32G and S33A). This indicates that *A. paludum* may considerably improve its sliding performance by using asymmetric rather than symmetric gait, especially for fast sliding (electronic supplementary material, figure S33A).

**Figure 5.**
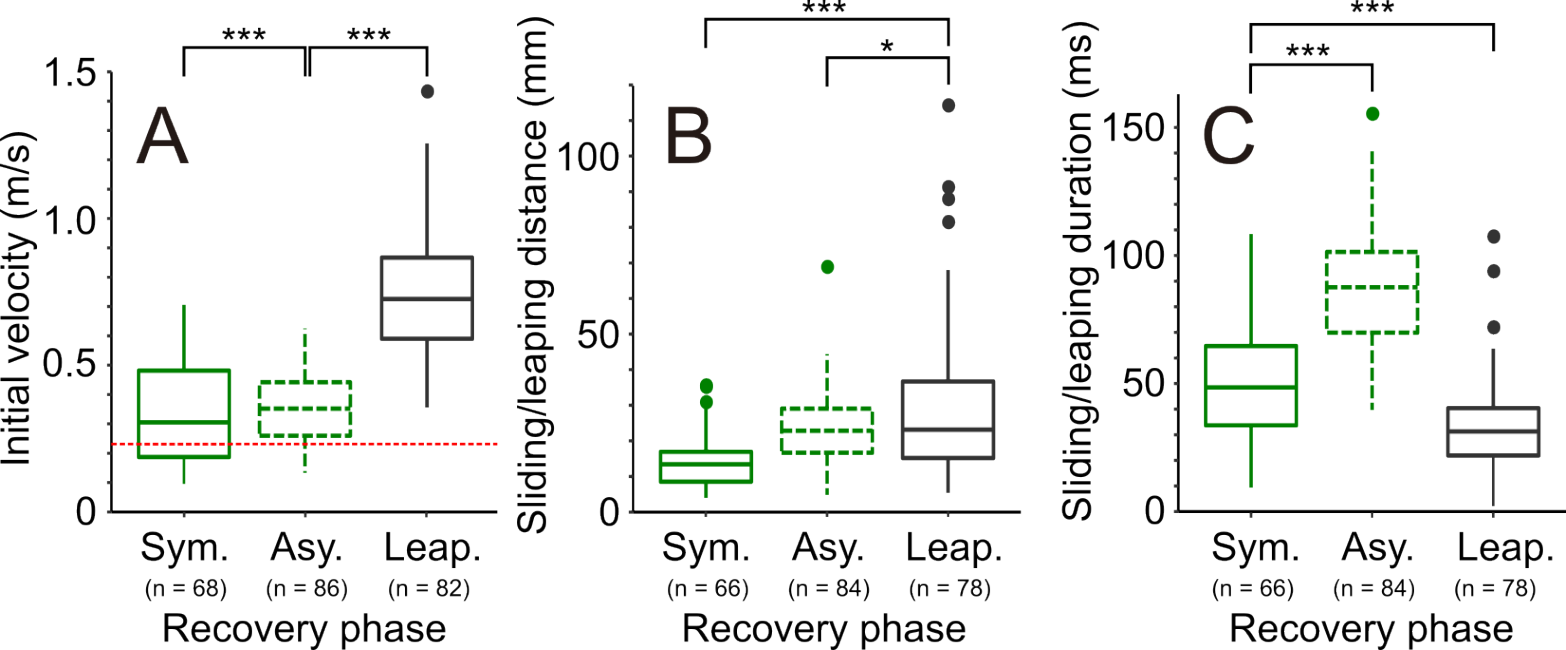
Velocity, distance and duration of three gait types observed in *A. paludum*. (A) Initial velocity, (B) sliding/leaping distance and (C) sliding/leaping duration in a recovery phase. Symmetric sliding, asymmetric sliding and leaping are marked as green solid, green dashed and grey solid lines, respectively. Critical velocity of wave-making, c=0.231, is marked with red dashed line in (A). The statistical analyses for (A–C) are in electronic supplementary material, tables S4–S6, and additional results are in electronic supplementary material, figures S27, table S7.

Observations of *A. paludum* revealed that individuals switched between symmetric and asymmetric gaits: symmetric propulsion followed by leaping occurred mostly at high body velocities (velocity at the end of propulsion > 0.5 m s^−1^; figure 5A, electronic supplementary material, figure S12; statistics in electronic supplementary material, table S4). Symmetric sliding was used over a relatively wide range of initial sliding velocities including relatively fast and relatively slow sliding (figure 5A). However, most of the asymmetric sliding occurred at the velocities that were larger than the theoretical threshold (c= 0.231 m s^−1^; red dashed line in figure 5A), above which capillary-gravity wave resistance starts to slow down the insects, especially during symmetric sliding. This leads to strong deceleration for symmetric compared to asymmetric sliding, which is especially pronounced at the velocities of 0.4−0.5 m s^−1^ (electronic supplementary material, figures S29B,G, S30B,G and S33A); 75% of asymmetric sliding (*n* = 65 out of 86 sliding events in *A. paludum*) occurred at the initial velocities higher than 0.258 m s^−1^ (lower quartile in figure 5A; electronic supplementary material, figure S33), when symmetric sliding already results in clearly stronger deceleration compared to the asymmetric sliding (electronic supplementary material, figure S33A). The lower deceleration in asymmetric sliding is associated with larger sliding distance (figure 5B; electronic supplementary material, table S5) and longer sliding duration (figure 5C; electronic supplementary material, table S6). However, these differences in distance and duration seem also affected by the animal directly: the end of sliding appears determined by the moment at which water strider ‘decides’ to put down its midlegs on the water to stop the sliding (electronic supplementary material, video S3).

Observations of the smaller species, *G. latiabdominis* (figure 2C1), also revealed some degree of behavioural plasticity: *G. latiabdominis* tended to use leaping at higher body speeds, thereby avoiding contact with surface which would create high-resistance (electronic supplementary material, table S3). However, this smaller species did not use asymmetric gait (figure 2C1), which coincides with a very small potential benefit of switching from the ancestral symmetric to the asymmetric gait as the difference in resistance/deceleration between these gaits is small for small-bodied species (electronic supplementary material, figures S31A,F and 32A,F; double arrowed black vertical lines) compared to the medium-sizes species (electronic supplementary material, figures S31B,G and S32B,G).

## 3. Discussion

Our theoretical analysis predicts that the small water striders (approx. 10 to 30 mg) from all six combinations of the three leg geometries and the two gait types can float during sliding. Although the symmetric gait by water striders with ‘short-wetted-foreleg geometry’ is theoretically possible (i.e. water striders can stay afloat), this can be performed only in a narrowly constrained area of light body mass or slow sliding in the small/medium-sized water striders, because if the body is heavy and/or locomotion is fast, the short wetted forelegs cannot create sufficient force to support the anterior body section on the water surface during sliding. Additionally, if small insects with intermediate-wetted-foreleg geometry do not orient their foreleg tarsi near-parallel to sliding direction, they may more easily break the water surface in fast sliding, which was observed in some cases of symmetric sliding of *A. paludum*. Downward force on anterior legs caused by anterior body downward rotation to restore original body pitch after the propulsive phase [26,48] may potentially contribute to surface breaking by anterior legs, albeit it has not been studied. Our model did not consider this phenomenon. If it happens then it will likely narrow down the area of floatability conditions to some extent, resulting in slightly more conservative predictions of floatability polygons.

The species with ‘intermediate-wetted-foreleg’ geometry seem to use a specific behavioural strategy that may prevent the meniscus breaking by forelegs during symmetric gait: ‘reversed foreleg’ position (electronic supplementary material, figure S17, video S9). *A. paludum* would sometimes bend their forelegs downward at coxa and femur-tibia joints resulting in foreleg tip pointing posteriorly and dorsal side of tibia+tarsus interacting with water surface while legs are parallel to the body movement. This can provide longer wetted leg to support the anterior body compared to the standard support by ventral surface of tarsus only. It was observed at high velocities when anterior normal forces are predicted to be especially high indicating that water striders’ use behavioural plasticity to adapt to conditions during each stride. The switching from normal to ‘reverse’ position may also help to reduce resistance during sliding, because when an insect changes tarsus direction during fast movement, the water-interacting parts of foreleg move backwards (during switching) while body moves forward, resulting in foreleg tarsus remaining in the same position relative to the water surface (i.e. foreleg does not create resistance, electronic supplementary material, video S9). Additionally, the elongation of the functional wetted length can decrease resistance similarly to the decrease in foreleg resistance due to tarsus elongation in the ‘long-wetted-foreleg’ species such as *P. tigrina* (electronic supplementary material, video S4). Future experiments should quantitatively evaluate the use of this strategy by *A. paludum*.

As the theoretically feasible combinations differ in resistance and deceleration, and as water striders seem to pay attention to the resistance (indicated by our observations of the behavioural plasticity in *A. paludum*, where the insects appeared to adaptively choose gait type), we hypothesize that natural selection or adaptive behavioural plasticity towards decreasing resistance may in certain conditions cause evolutionary or behavioural shifts from the ancestral [30,39,40] symmetric gait of water striders with ‘intermediate-wetted-foreleg geometry’ towards either the asymmetric gait or ‘long-wetted-foreleg geometry’.

Asymmetric gait substantially decreases resistance and deceleration, but it involves weaker thrust from only one midleg aided by contralateral hindleg. Hence, it may be feasible only in habitats where frequent strong strokes are less important (stagnant or slow-flowing water). Long-wetted-foreleg geometry reasonably decreases resistance (longer forelegs create lower resistance) while maintaining high thrust from two symmetrically pushing midlegs, which may be especially important when frequent rowing with high thrust is highly beneficial (e.g. in fast flowing water). The presence of ‘long-wetted-foreleg geometry’ (and apparently also the symmetric gait) even in the small-sized taxa typical for fast current (e.g. *Metrocoris* [30]) or for turbulent oceanic waters (*Halobates* [22,37]) is consistent with the idea that ‘long-wetted-foreleg geometry’ is advantageous in turbulent habitats.

When body mass reaches the range represented by *P. tigrina* and *G. gigas* (range of about 80−500 mg), water striders with typical ‘intermediate-wetted-foreleg geometry’ would not be able to support their bodies on the surface during symmetric sliding (when the anterior body mass is supported by two forelegs). The model predicts, and literature [45] suggests (electronic supplementary material, figures S2 and S3), that there are two solutions. One solution involves a shift to ‘long-wetted-foreleg geometry’ by the elongation of forelegs (recent studies in the genetics of morphology in Gerridae identified genes that may be involved in the leg elongation [49,50]) while maintaining the standard symmetric gait like in Ptilomerinae. Another solution involves asymmetric gait, like in *G. gigas*. The difference between *G. gigas*, which lives in slower flowing waters, and *P. tigrina*, which lives in faster flowing water, is consistent with the idea that even though the asymmetric sliding always creates less resistance and does not cause meniscus breaking regardless of body mass and leg geometry, *P. tigrina* does not use the asymmetric sliding (exception shown in electronic supplementary material, video S5) because of the importance of strong thrust in the very frequent short strides against fast flowing water in their habitat [44] (see also electronic supplementary material, video S4). Similar to *G. gigas*, we observed that *Cylindrosthetus costalis* with ‘short-wetted-foreleg geometry’ and large body size (electronic supplementary material, figure S2A) also shows asymmetric gait in its natural habitat (electronic supplementary material, video S8). Sliding performance in asymmetric gait does not depend on the relative wetted foreleg length. Therefore, in accordance with the rules of competition among developing body parts [51], it is associated with shortening of the wetted forelegs that are no longer needed for support during sliding. Finally, as already speculated [38], the asymmetric gait is associated with asymmetry in thrust (stronger on the side of the pushing midleg than on the side of the midleg stretched forward), which leads to torque in the horizontal plane. Therefore, the especially elongated wetted hindlegs characterizing the ‘short-wetted-foreleg geometry’ (electronic supplementary material, figure S2C) of the asymmetrically striding species may play a role as a rudder preventing rotation of body axis. If this is correct, then hindlegs in heavy asymmetrically striding species serve two locomotory functions, adding to the thrust and counteracting the torque.

Based on the results, we propose that habitat type may affect evolutionary trajectories shaping the wetted leg geometry as water striders evolve large body. This would lead in large water striders to the asymmetric gait in species from slow-flowing waters or to the long-wetted-foreleg/symmetric gait combination in species from fast currents. The requirements for frequent and strong propulsion in fast currents may additionally trigger evolution of special micro-structures for rowing [37,44,52] and the associated loss of the midlegs’ function of supporting the water strider on water surface [22,44]. If these hypotheses are correct, then Gerridae illustrate how physical environment channels the morphological and behavioural evolution towards either of the two physically feasible adaptive solutions for locomotion by large-sized water striders. We also hypothesize that predators may additionally affect this ‘channeling’ of morphological and behavioural evolution. The ‘zig-zagging’ asymmetric gait movements may provide better protection from tracking by predators than the near linear and more predictable movements in symmetric striding, but only in slow flowing/stagnant waters where detection of vibrations and waves on quiet surface is easier than in habitats with fast flowing waters where surface turbulence may camouflage the waves created by an insect.

In summary, the model provides a solid theoretical basis for the next comparative step of research to understand the evolution of allometry of gait in water striders. It also provides insights into bio-inspired engineering of water walking robots of various sizes [15,27,32,53,54]. Another interesting research theme is the behavioural ontogeny of *G. gigas*. Since *G. gigas* uses asymmetric gaits due to their large body, juveniles of *G. gigas* with lighter bodies may either have different leg geometry or use symmetric gaits. A similar experiment can be performed by adding artificial weight to *A. paludum*, which uses all types of gaits, to observe how their behavioural plasticity is affected by different body weight. Information currently available in the literature (see caption to electronic supplementary material, figure S2) allowed us to present only a preliminary view of the relationship between body length and ‘wetted leg geometry’ (electronic supplementary material, figures S2 and S3), which confirmed the model predictions. Once solid morphological and behavioural data on gait types in natural habitats across a variety of species of different sizes are collected and/or confirmed, the predictions can be properly tested in quantitative comparative phylogeny-based analyses in Gerridae.

## 4. Material and methods

### (a) Measurements, observations and experiments

We measured body mass (with 0.1 mg precision) and morphological variables (from photographs using ImageJ; https://imagej.nih.gov/ij/) described in electronic supplementary material, figure S13 for six species: *G. latiabdominis* (*n* = 16; Seoul, Korea), *A. remigis* (*n* = 6; Huyck Preserve, USA), *G. gracilicornis* (*n* = 16; Seoul, Korea), *A. paludum* (*n* = 21; Seoul, Korea), *Ptilomera tigrina* (*n* = 18; Me Linh Station for Biodiversity, Vietnam) and *G. gigas* (*n* = 25; Pu Mat National Park, Vietnam). The wetted leg was assumed to be tarsus of foreleg, tarsus+tibia of midleg and hindleg. Leg diameter was measured including hair around the leg [20].

We filmed *G. gigas* and *P. tigrina* in their natural habitats (standard and high-speed movies at 250, 500 and 1000 fps), and *P. tigrina*, *A. paludum* and *G. latiabdominis* in acrylic containers filled with water (standard and high-speed at 1000 fps) with Sony RXIII-10 camera. A total of 50 striding events of *G. gigas* and 12 striding events by *P. tigrina* were filmed and used to determine their gait type, and a total of 236 striding events from six individuals of *A. paludum* and 13 striding events from five individuals of *G. latiabdominis* were digitized. The high-speed videos that were shot directly from above the water strider with scale at the level of the water surface were digitized and analysed using Tracker program (https://physlets.org/tracker/) to determine body velocity and acceleration. For analysis of striding events on the flowing current (i.e. strides in natural habitats of *P. tigrina* and *G. gigas*; electronic supplementary material, figures S8–S11, S23−S26, S31 and S32), we adjusted coordinates of a water strider in each frame by considering average flow velocity vector of flowing creek at each location where insects strode. The average flow velocity vector was calculated from digitized frame-by-frame movements of an object flowing across the location where an insect was filmed (e.g. bubble, small leaf, electronic supplementary material, figure S10). Every movie for digitization was captured perpendicular to the water surface, including *G. gigas* strides in their natural habitats, except for one movie of *P. tigrina* in the creek (electronic supplementary material, figures S8 and S23). This movie was only used to show velocity trend of *P. tigrina* in their natural habitats, and movies from container were used for a more detailed analysis (electronic supplementary material, figures S11,22) in this species. For the observation of foreleg tarsus orientation in symmetric sliding, we filmed 79 closed-up high-speed videos from five individuals of *A. paludum*. We categorized anterior leg orientation as parallel, diagonal, orthogonal, reversed and noted if meniscus breaking occurred (sample sizes in electronic supplementary material, table S2).

For statistical comparisons of body velocity between different gait types in *G. latiabdominis* (*n* = 8 striding events by four individuals), we used Wilcoxon signed-rank test (https://astatsa.com/WilcoxonTest/, https://www.aatbio.com/tools/mann-whitney-wilcoxon-signed-rank-test-calculator). We used 8 paired striding events out of 13 digitized events for the non-parametric test (5 unpaired events were excluded). For statistical comparisons of initial body velocity of recovery phase (i.e. start of passive sliding phase) among three locomotion modes (three gait types) of *A. paludum* (*n* = 236 striding from six individuals), we used *lmerTest* and *gamlss* packages (R v. 3.6.1). The distance traveled and the duration of recovery phase (i.e. the passive sliding phase) were also analysed in a similar manner, but only for those events (*n* = 228 striding events from six individuals) that were naturally ended by the water strider itself touching the water surface with their midleg(s) (e.g. excluding sliding events that ended by hitting the container’s wall).

Additionally, we chose 72 striding events of *A. paludum* that have recovery phase duration long enough (50−80 ms) to empirically evaluate the deceleration and subsequently the resistance. These data were analysed using the general additive model (*gamlss* package in R). Finally, for a small subset of striding events (8 for *G. latiabdominis*, 16 for *A. paludum*, 8 for *P. tigrina* and 5 for *G. gigas*), we digitized the locomotion from high-speed movies in a frame-by-frame manner in order to extract information for evaluation of acceleration and force generated during propulsive phase of each species (electronic supplementary material, figure S26).

### (b) Numerical calculations based on the model

An essential description of the model is presented in §2, and the full description of the model and numerical calculations is presented in electronic supplementary material 1, parts 3 and 4. Based on the mathematical model, we built a computational model in MATLAB. Figures showing model output were also prepared using MATLAB.

## Data Availability

Datasets of species measurement associated with analyses/figures and MATLAB codes for the theoretical model are deposited at Zenodo [55]. Supplementary material is available online [56].
